# The Faecal Microbiome of the Wild European Badger *Meles meles*: A Comparison Against Other Wild Omnivorous Mammals from Across the Globe

**DOI:** 10.1007/s00284-022-03064-4

**Published:** 2022-10-17

**Authors:** James F. Scott-Baumann, Jessica C. A. Friedersdorff, Bernardo Villarreal-Ramos, Jonathan King, Beverley Hopkins, Richard Pizzey, David Rooke, Glyn Hewinson, Luis A. J. Mur

**Affiliations:** 1 Institute of Biological, Environmental and Rural Science, Aberystwyth University, Aberystwyth, SY23 3DA Ceredigion UK; 2 Centre of Excellence for Bovine Tuberculosis, Aberystwyth University, Aberystwyth, SY23 3AR Ceredigion UK; 3 Wales Veterinary Science Centre, Y Buarth, Aberystwyth, SY23 1ND Ceredigion UK; 4 ProTEM Services Ltd, Horsham, West Sussex UK; 5 TB Research Group, 5Animal and Plant Health Agency, New Haw, Addlestone, KT15 3NB Surrey UK

## Abstract

Here we investigate the faecal microbiome of wild European badgers *Meles meles* using samples collected at post-mortem as part of the All Wales Badger Found Dead study. This is the first published characterisation of the badger microbiome. We initially undertook a sex-matched age comparison between the adult and cub microbiomes, based on sequencing the V3–V4 region of the 16S rRNA gene. Analysis used the QIIME 2 pipeline utilising DADA2 and the Silva database for taxonomy assignment. Fusobacteria appeared to be more abundant in the microbiomes of the cubs than the adults although no significant difference was seen in alpha or beta diversity between the adult and cub badger microbiomes. Comparisons were also made against other wild, omnivorous, mammals’ faecal microbiomes using publicly available data. Significant differences were seen in both alpha and beta diversity between the microbiomes from different species. As a wildlife species of interest to the disease bovine tuberculosis, knowledge of the faecal microbiome could assist in identification of infected badgers. Our work here suggests that, if comparisons were made between the faeces of bTB infected and non-infected badgers, age may not have a significant impact on the microbiome.

**Supplementary Information:**

The online version contains supplementary material available at 10.1007/s00284-022-03064-4.

## Introduction

Bovine tuberculosis (bTB) represents a huge economic cost to UK cattle farming. Badgers are known to carry the causative organism, *Mycobacterium bovis*, and can pass it to farmed cattle [1]. Current tests available for diagnosis of bTB in badgers are limited by sensitivity and practicality (requiring trapping live animals) [2]. In humans, changes in the gut microbiome have been shown to occur with tuberculosis infection [3]. These changes in the gut microbiome may hold potential diagnostic purposes that could then be used on faecal samples easily collected from around setts, avoiding trapping of live animals.

However, before assessing the badger’s faecal microbiome changes associated with bTB infection, there must be an understanding of the healthy microbiome and the associated sources of variation. Age has been shown to the one of the most significant factors affecting the microbiome in animals like it has in humans, with maturation of the microbiome over time from birth to adulthood [4, 5]. In this study, a comparison is first made between the faecal microbiomes of adult and cub (<1 year old) badgers collected during post-mortem as part of the ‘All Wales Badger Found Dead’ project. Secondly, a comparison is made between these badger microbiomes and 24 other faecal microbiomes from different wild, omnivorous mammal species collected from the environment from across the globe [6].

## Materials and Methods

### Badger Sample Collection and Processing

The badgers involved in this study were collected dead on the side of the road as part of an ongoing surveillance study by Welsh Government and the Animal Plant Health Agency (APHA); the All Wales Badger Found Dead study. Badgers were collected and brought to a laboratory where they were deemed appropriate for post-mortem (PM) if they were intact, not distended with gas, with no severe myiasis and were not frozen. Carcasses were stored for less than 4 days at 4 °C before PM. PM involved an external examination; including weighing, measuring, sexing, approximate aging based on dental wear, and checking for lactation if female. Badgers were scanned for microchips as well as clipping of guard hairs or any colour marker to indicate historical trapping and vaccination. Any signs of external injury, bite wounds, illegal trapping or snaring were also noted. Internal examination was focussed on identification of any gross lesions and samples of tissues were collected for mycobacterial culture. Detailed examination was made of the pericardial sac, lungs, liver and kidneys including internally by making several, longitudinal incisions across each. Lymph nodes were incised at least once and examined for lesions [submaxillary, retropharyngeal, external cervical, axillary, bronchial, mediastinal, hepatic, gastric, renal (when located), mesenteric, internal iliac, external iliac, superficial inguinal, popliteal]. Two pools of samples were then created; pool one contained retropharyngeal, bronchial lymph nodes, mediastinal and hepatic lymph node samples, and pool two contained a section of any bite wound or any internal visible lesions suggestive of bTB. The samples were preserved in 1% aqueous cetylpyridinium chloride and are posted to the APHA laboratory in Starcross, (Devon, UK) for bTB testing. At Starcross, the samples are washed in sterile 0.85% saline, then homogenized and inoculated onto six modified Middlebrook 7H11 agar slopes and incubated at 37 °C for up to 12 weeks. Any bTB that was grown was sent to APHA Weybridge for genotyping. Culture positivity and genotyping provided the basis for designation of badgers as positive for bTB. At the time of PM, a faecal sample was collected using a sterile spatula from the final 5 cm of the rectum, so as to closely mimic a faecal sample as possible. This was then frozen at –80 °C until further analysis. Only samples from bTB negative badgers were used for this study. For this study a comparison was made of the effect of age on the badger’s microbiome by comparing six cubs and six adults, these were age-matched and all were male.

## Intra-species Age Comparison of Badger Microbiomes

An aliquot of 0.2 g (±0.02 g) of faeces was taken and the genomic DNA was extracted using a FastDNA SPIN kit for soil (MP Biomedical, Santa Ana, USA) following manufacturer’s instructions. Bead beating was carried out in a FastPrep-24 machine (MP Biomedical, Santa Ana, USA) with three 30 s cycles at speed setting 6.0 for seconds, with cooling on ice for 30 s between cycles. A blank consisting of no sample but kit reagents only was included to identify potential contamination or kit-ome effects. Following DNA extraction the Illumina MiSeq platform was used to amplify the V3–V4 region of the 16S rRNA [7]. Read quality for the newly generated data and the downloaded data was reviewed using FastQC and MultiQC. All downstream analysis of the raw read files was done using the QIIME 2 pipeline (QIIME2 v2021.4 [8]). Primers were trimmed from reads, and forward and reverse reads were trimmed when PHRED score dipped below 20. Sampling depth was cut-off at 3816 reads in order to keep the sample with the lowest number of reads (fat dormouse). Rarefaction curves suggested minimal loss of diversity at this cut-off (Fig. S1). Taxonomy was assigned to OTUs using the Silva database.

## Inter-species Comparison of Wild Omnivore Microbiomes

To provide a comparison of faecal microbiomes from other wild mammals, samples were downloaded that were generated from the Youngblut et al. [6] study (European Nucleotide Archive, study Accession Number PRJEB29403). All those used were faecal microbiomes from omnivorous, wild mammals (*n* = 24) and were generated using primers for just the V4 region of the 16S rRNA.

## Results and Discussion

### Intra-species Age Comparison of Badger Microbiomes

**Fig. 1:**
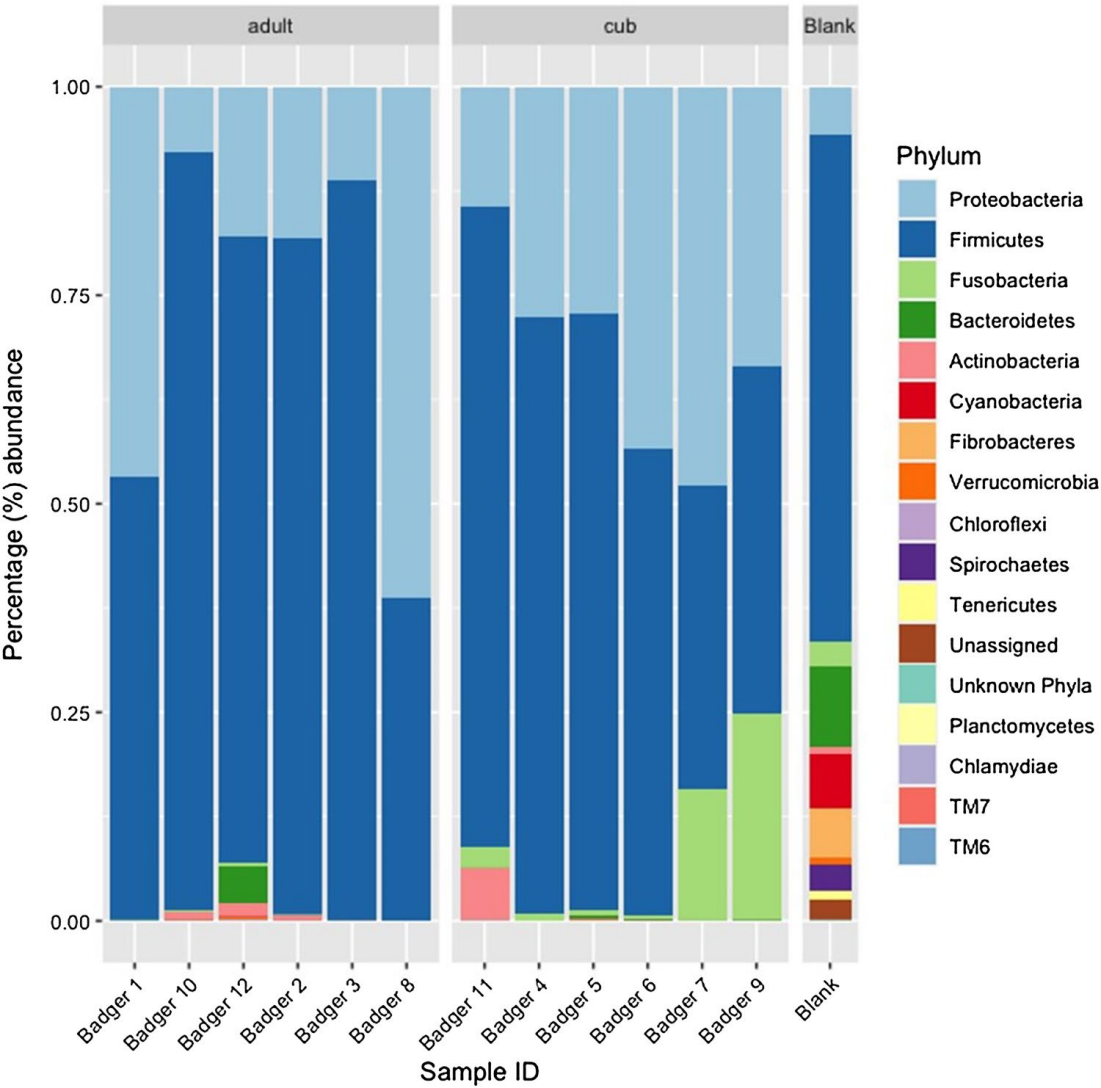
Percentage abundances of different phyla of bacteria present in the faecal microbiome of adult and cub badgers (*n* = 12)

More than 99.9% of the operational taxonomic units (OTU) were assigned to known phyla using the SILVA database for both the adults and the cubs. The most predominant bacterial phyla present in the badgers’ faecal microbiomes were Proteobacteria and Firmicutes, together accounting for 75% or more of the percentage abundance across all samples (Fig. 1). This contrasted with other human and animal studies which have often shown a typical predominance of Firmicutes and Bacteroidetes [9, 10]. Fusobacteria appeared to be more abundant in the microbiomes of the cubs (mean ~7.5%) than the adults (mean ~0.1%). The “kit-ome” blank sample showed a wider diversity of phyla suggesting that this was not a major source of contamination bias in the badger faecal microbiomes.

At the genus level the proportion of OTUs assigned for adults and cubs were 68.9 and 54.2%, respectively. At the species level only 7.7 and 6.5% of OTUs were linked to uncultured species or those identified from previous metagenomes, for adults and cubs, respectively. Of the OTUs successfully identified using SILVA taxonomy, *Romboutsia hominis*, and genera *Shigella*, *Clostridium sensu stricto 1*, *Paeniclostridium* and *Terrisporobacter* were common to all badger samples. There were no species or genera found to be unique to all cub or to all adult samples.

**Fig. 2:**
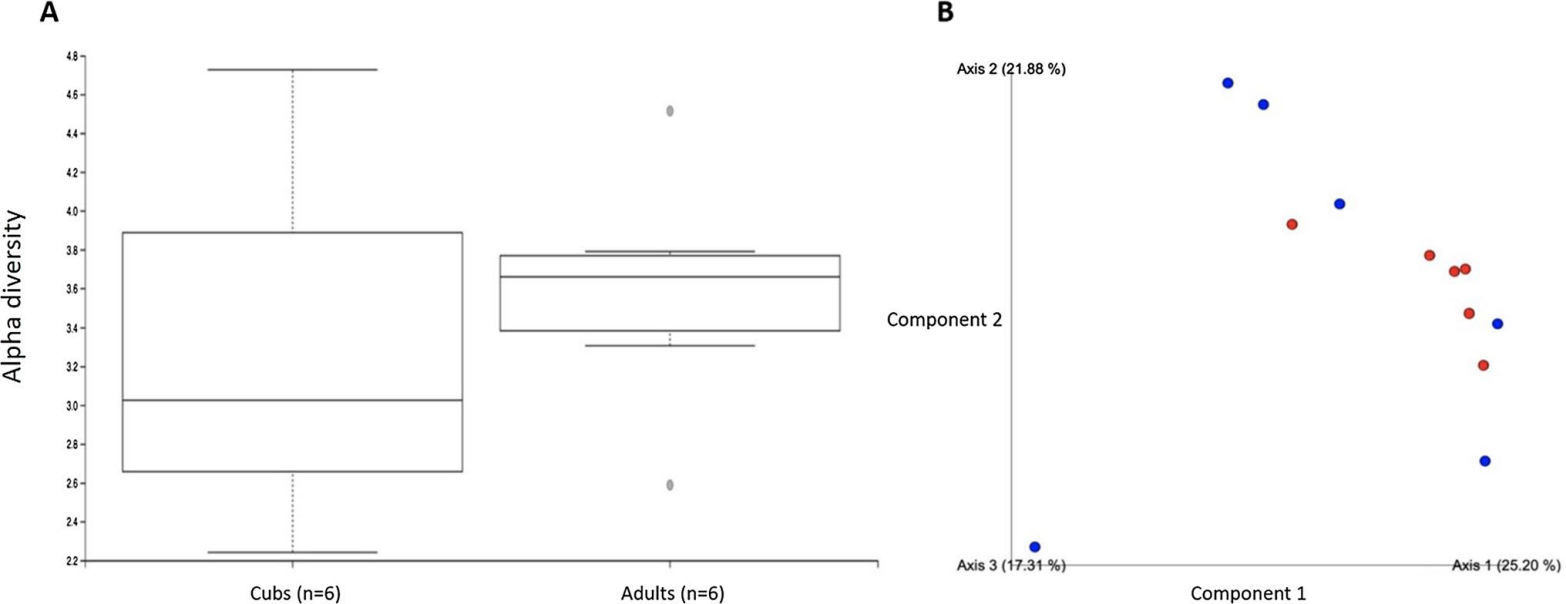
Diversity estimates of the different bacterial genera in the badger microbiome based on **A** alpha diversity (Shannon index) and **B** beta diversity (Bray–Curtis dissimilarity) comparing cubs (red) and adults (blue) showed no significant differences between groups (Color figure online)

Alpha diversity comparisons (Shannon and Simpson’s index) at genus level showed no significant differences (*P* = 0.42 each) between the two age groups using Kruskal–Wallis pairwise (Fig. 2a). Beta Diversity comparisons (Bray–Curtis, Jaccard, unweighted Unifrac, weighted Unifrac) at genus level showed no significant differences (*P* = 0.14, 0.71, 0.19, 0.13 respectively) between the two age groups using PERMANOVA (Fig. 2b). This indicated that the adult and cub badgers’ faecal microbiomes were similarly diverse in our sample populations.

Age has previously been shown in animal models, like in humans, to be a significant factor in the faecal microbiome, in its development and maturation over time [4, 5]. Given that the only cubs recruited into this study were old enough to be free roaming and leave the sett, they may have already developed a ‘mature’ microbiome, when compared to those younger cubs in the setts. This would not be captured in this study by the nature of the sampling methods. Knowing whether a faecal microbiome changes with age is key when going on to look for marker species present in faeces as an indicator for bTB infection.

## Inter-species Comparison of Wild Omnivore Microbiomes

**Fig. 3:**
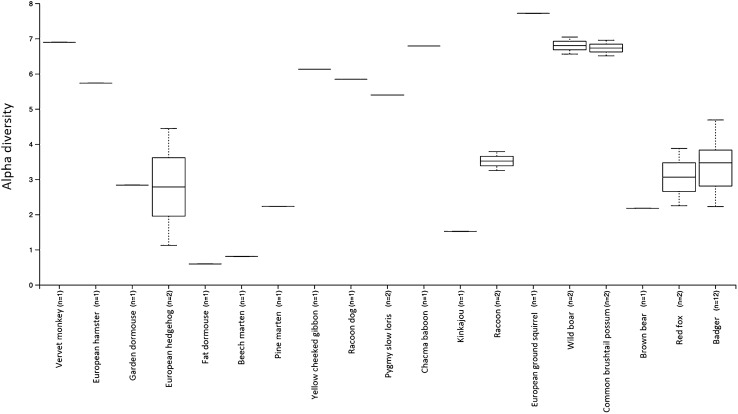
Comparison of alpha diversity using the Shannon index for genera observed in the microbiome across all the omnivorous mammals compared in this study

**Fig. 4:**
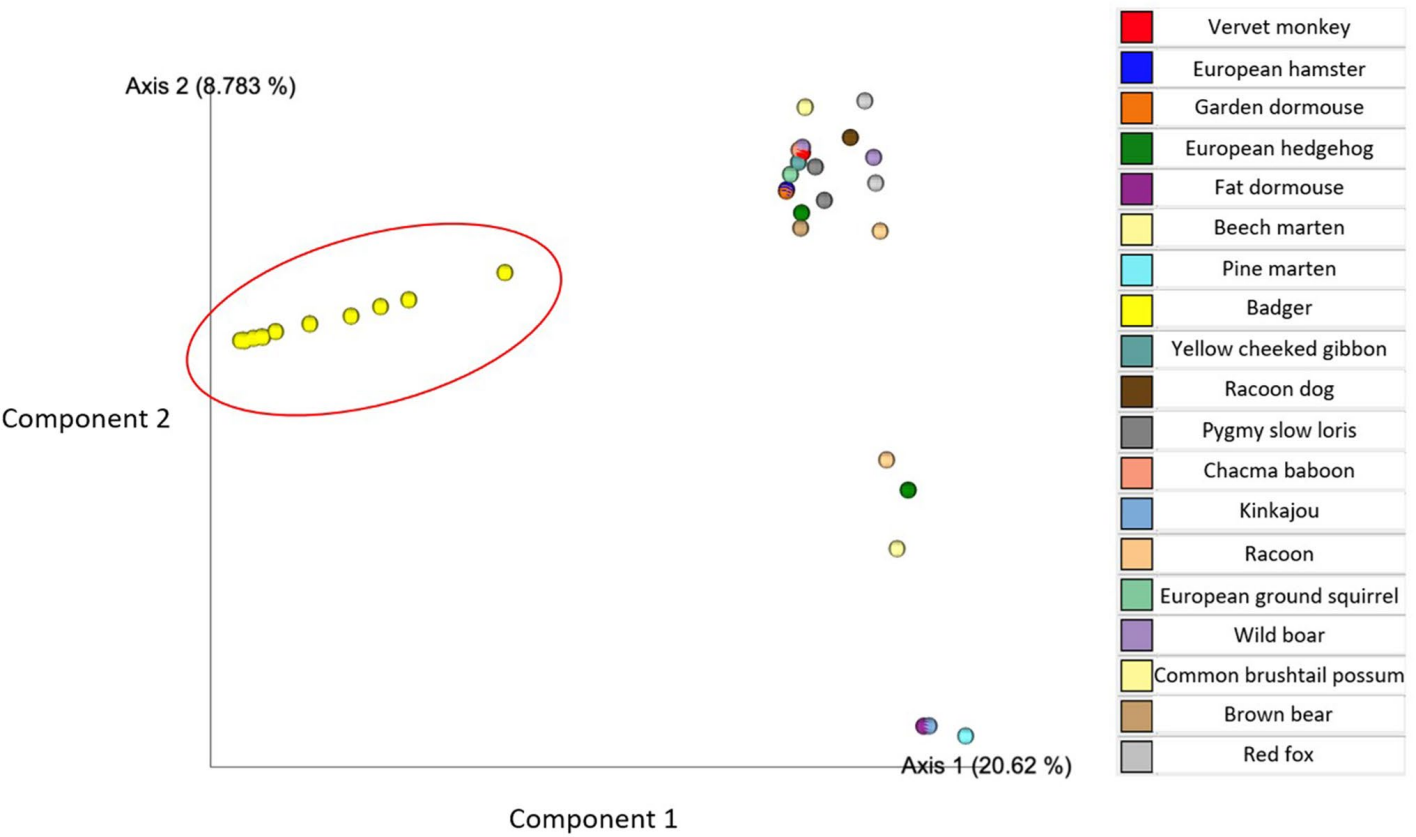
Principal coordinates analysis showing beta diversity, based on the Bray–Curtis dissimilarities, at genus level. European badgers *Meles meles* (yellow, *n* = 12 and indicated by an open red circle which has no mathematical significance) separate clearly as a group (Color figure online)

**Fig. 5:**
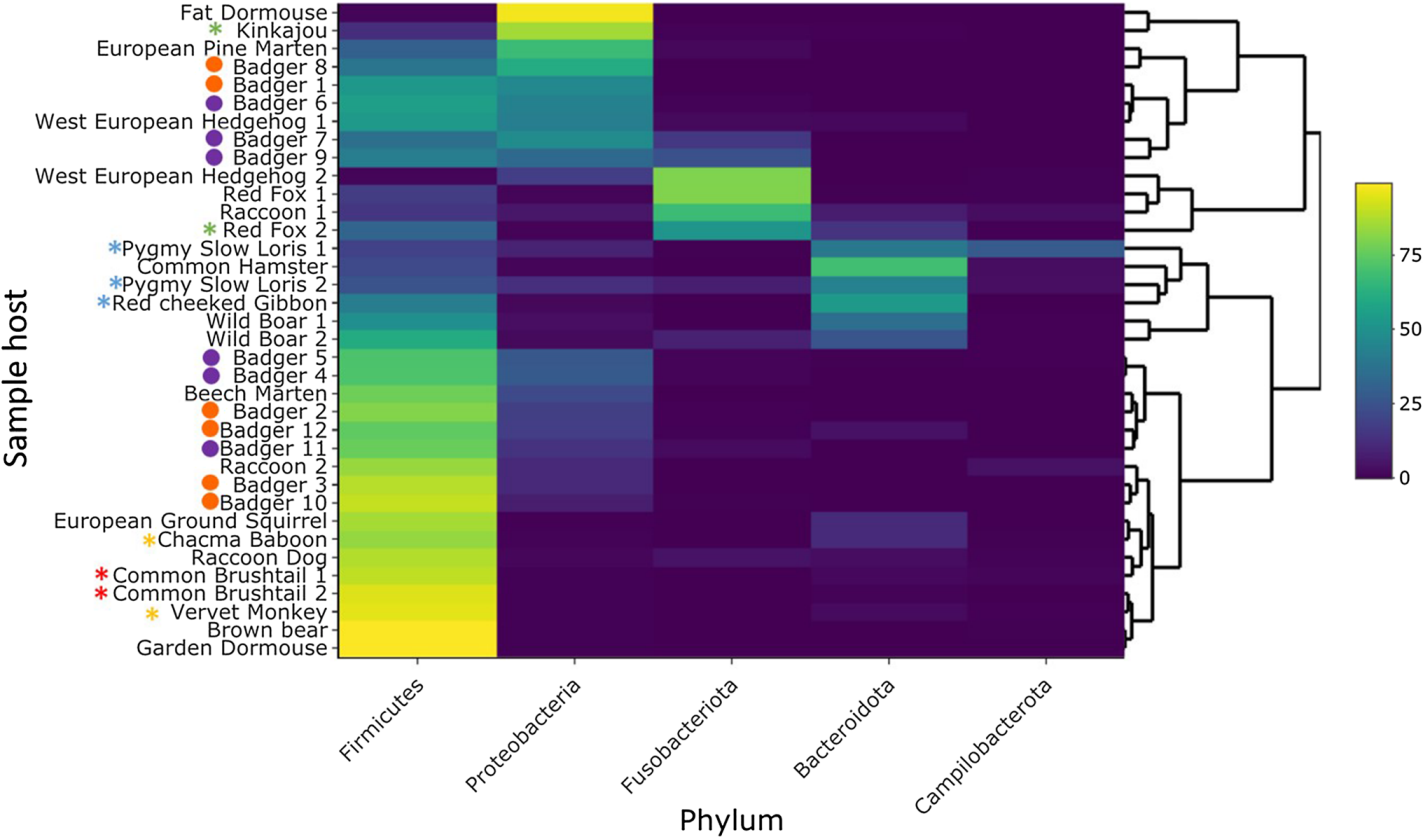
Percentage abundance of bacterial phyla present in different omnivorous mammals’ microbiomes. Only phyla contributing 25% or more to a host’s microbiome are displayed. Colours in the heatmap correspond to these legend on the right, with yellow 100% and purple 0% percentage abundance. Purple circles denote badger cubs and orange circles denote badger adults. Asterisks denote country sampled from (green = Costa Rica, blue = Vietnam, yellow = South Africa, red = Australia) (Color figure online)

The percentage abundances of all bacterial phyla identified across all samples shows that Firmicutes, Proteobacteria, Fusobacteria and Bacteroidetes were the most prevalent (Fig. S2). Alpha diversity comparisons showed that badger faecal microbiomes were more diverse than other members of the family Mustelidae (beech marten, pine marten), but were less diverse than other mammals such as the European ground squirrel, which had the highest alpha diversity. There were significant differences between faecal microbiome diversity at the order (*P* = 0.005), family (*P* = 0.005) and genus (*P* = 0.04) levels of mammal host groupings with the Shannon diversity metric (Fig. 3). Beta Diversity comparisons (Bray–Curtis, Jaccard, unweighted Unifrac, weighted Unifrac) at the genus level all showed significant differences between the different hosts at the level order (*P* < 0.001), family (*P* < 0.001) and genus (*P* < 0.001) using PERMANOVA (Fig. 4). Differences in the percentage abundance between the most prevalent bacterial phyla identified in the microbiomes are displayed using hierarchical clustering (Fig. 5). A minimum percentage abundance cut-off of 25% was used: any phyla contributing less than 25% percentage abundance to any mammal microbiome have been removed.

Several studies have shown that host phylogeny and diet have significant impact on the faecal microbiome [11, 12], and captivity has also been shown to affect the microbiome markedly [13, 14]. From the Youngblut et al. [6] study only hosts that were omnivorous, wild and mammalian were selected, thereby allowing closer comparisons with the badgers. These 24 samples showed a predominance of Firmicutes followed by Bacteroidetes, which is in line with several other studies [9, 10], as compared to the badgers. This could be influenced by the badger samples being collected from the rectum of deceased animals which may have spent up to 4 days in cold storage. This time in cold storage was also additional to the time carcasses spent in the environment before they were seen and collected. In contrast to the other wild mammal samples that were collected as faecal samples from the field. However Choo et al. [15] found that refrigeration of faecal samples at 4 °C for 72 h before 16S rRNA analysis did not differ significantly from those stored immediately at –80 °C. Another potential confounding variable when comparing across the two studies is the slightly different areas of the 16 s region amplified; V3–V4 in this study and V4 in Youngblut et al. [6]. These differences in study design must be considered when interpreting the significant difference in beta diversity between the badgers and the other omnivorous mammals (Fig. 4).

Considering the differences between the faecal microbiomes (Fig. 5), the influence of diet is likely to be a major determinant. One particular group of host species, which includes the two red foxes, one West European hedgehog and one racoon, had higher levels of Fusobacteria, and formed a clade (see Fusobacteroita, Fig. 5). Several studies, both human and animal, have shown a proportional decrease in the ratio of Fusobacteria to Firmicutes associated with a higher fibre diet [16, 17]. This may be associated with the fact that species like *Fusobacterium varium* can catabolise amino acids as well as carbohydrates [18]. Red foxes, depending on what food is available with regards to the season, local habitat and proximity to humans, can be almost exclusively carnivorous [19]. West European hedgehogs can similarly be almost entirely insectivorous in some situations [20]. Racoons are highly opportunistic and their proximity to humans and access to anthropogenic food can affect their diet, so much so that it has resulting effects on their health [21]. One could hypothesise therefore that the animals in this group might have adopted a more carnivorous diet, which led to greater levels of Fusobacteria in their microbiome.

Another group, including both the wild boar, both pygmy slow loris, the red-cheeked gibbon and the common hamster, had much higher levels of Bacteroidetes than the rest of the samples. In both human and animal models Bacteroidetes can be altered with dietary changes; being positively associated with fat but negatively associated with fibre [22, 23]. Wan et al. [24] also found an increased abundance of Bacteroidetes with a high fat diet, with a simultaneous decrease in Firmicutes. However another study found increased proportions of faecal *Bacteroides* associated with a high carbohydrate/high glycaemic index (rapidly digested) diet, rather than high fat diets [25]. Diet clearly is significant factor for faecal microbiomes, and the diet of badgers has been shown to vary greatly, even within individuals in the same sett who therefore have access to the same dietary resources [26]. This could therefore explain the variability seen in the badgers’ microbiomes here.

Considering alternative influencing factors, the microbiomes could be influenced by geographical origins of sample. Most faecal samples came from Europe, a few coming from South Africa, Australia, Costa Rica and Vietnam. Figure 5 suggests that origins could influence the distribution of percentage abundance of Phyla present in animals’ faecal microbiomes. For example, all samples from Vietnam (the red-cheeked gibbon and the two pygmy slow loris samples) were closely located. Similarly, the microbiomes of the common brushtail possums (both from Australia) and the chacma baboon and vervet monkey (both from South Africa) appeared close together. The two raccoon samples, which are the only replicate samples (from the same species) that are not closely associated on the dendrogram, are from separate continents; Costa Rica (Racoon 1) and Austria (Raccoon 2).

Care must be taken when drawing conclusions about the individual microbiomes, given that for most host species from the Youngblut et al. [6] study they were presented by a single faecal sample from a single individual. The importance of this is indicated by the variation seen in our multiple assessments of badger microbiomes (*n* = 12). Indeed, the microbiome of some species can vary much more widely between individuals, when compared to other closely related species; such as hares and rabbits [10].

## Conclusion

This study is the first of its kind published on the faecal microbiome of wild European badgers. Despite the possible limitations of using post-mortem samples, it provides an initial understanding of the faecal microbiome for this population at post-mortem. Multiple studies have used post-mortem samples from badgers for monitoring of bTB [27, 28] and this work provides evidence that such samples could also be used for microbiome analysis, for instance in the comparison of *M. bovis* infected and non-infected badgers.

## Supplementary Information

Below is the link to the electronic supplementary material.

Supplementary file1 (TIF 393 kb)

Supplementary file2 (TIF 1137 kb)

## Abbreviation

bTB: Bovine tuberculosis

## Declarations

### Data Availability

The datasets generated during and/or analysed during the current study are at the National Centre for Biotechnology Information (NCBI) Sequence Read Archive (SRA) under BioProject ID: PRJNA777923, https://www.ncbi.nlm.nih.gov/sra/?term=PRJNA777923.
