# Supplementary figures and images for: The Faecal Microbiome of the Wild European Badger Meles meles: A Comparison Against Other Wild Omnivorous Mammals from Across the Globe
Source: Curr Microbiol. 2022 Oct 17;79(12):363. doi: 10.1007/s00284-022-03064-4 (PMC9576668; doi:10.1007/s00284-022-03064-4)

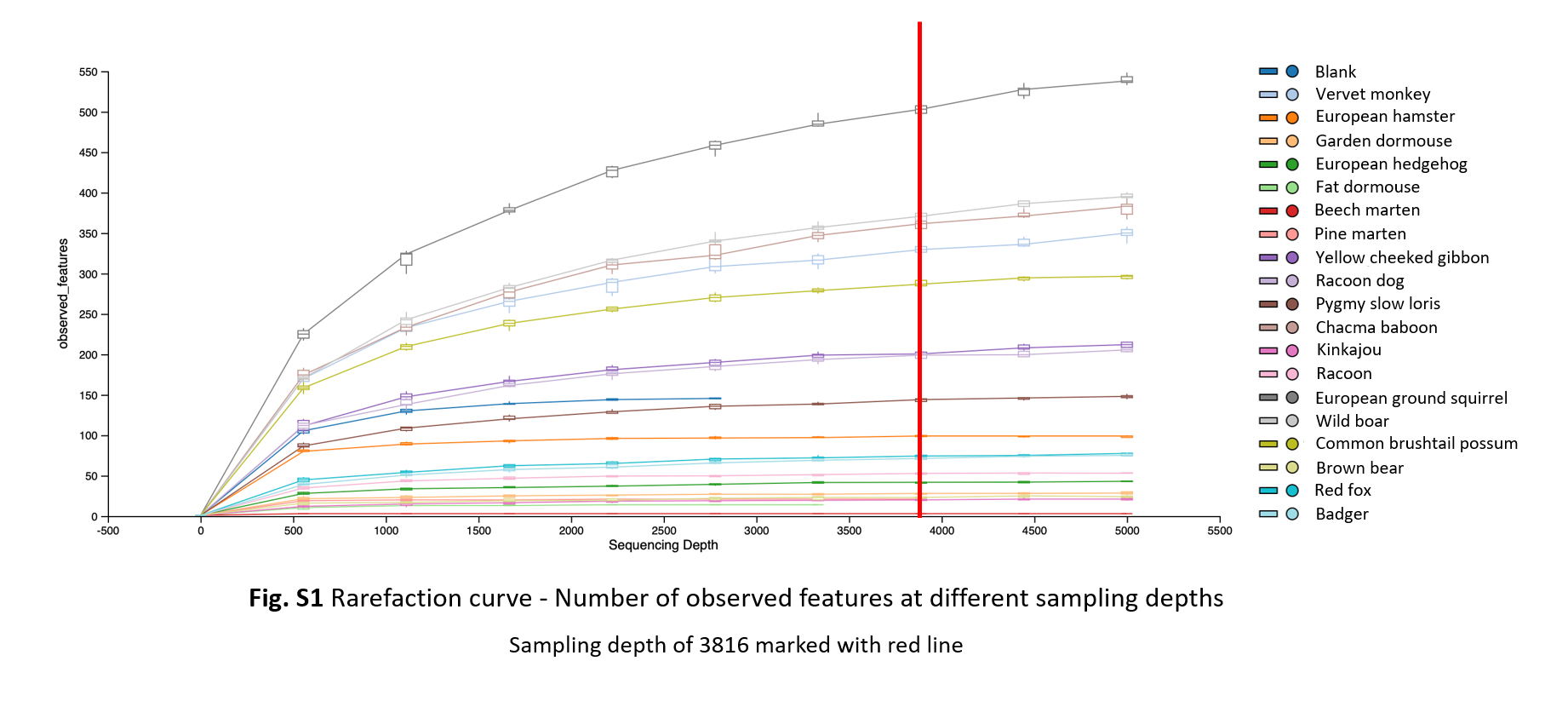

Supplement: Supplementary file 1 — Supplementary file1 (TIF 393 kb) [file 284_2022_3064_MOESM1_ESM.tif]

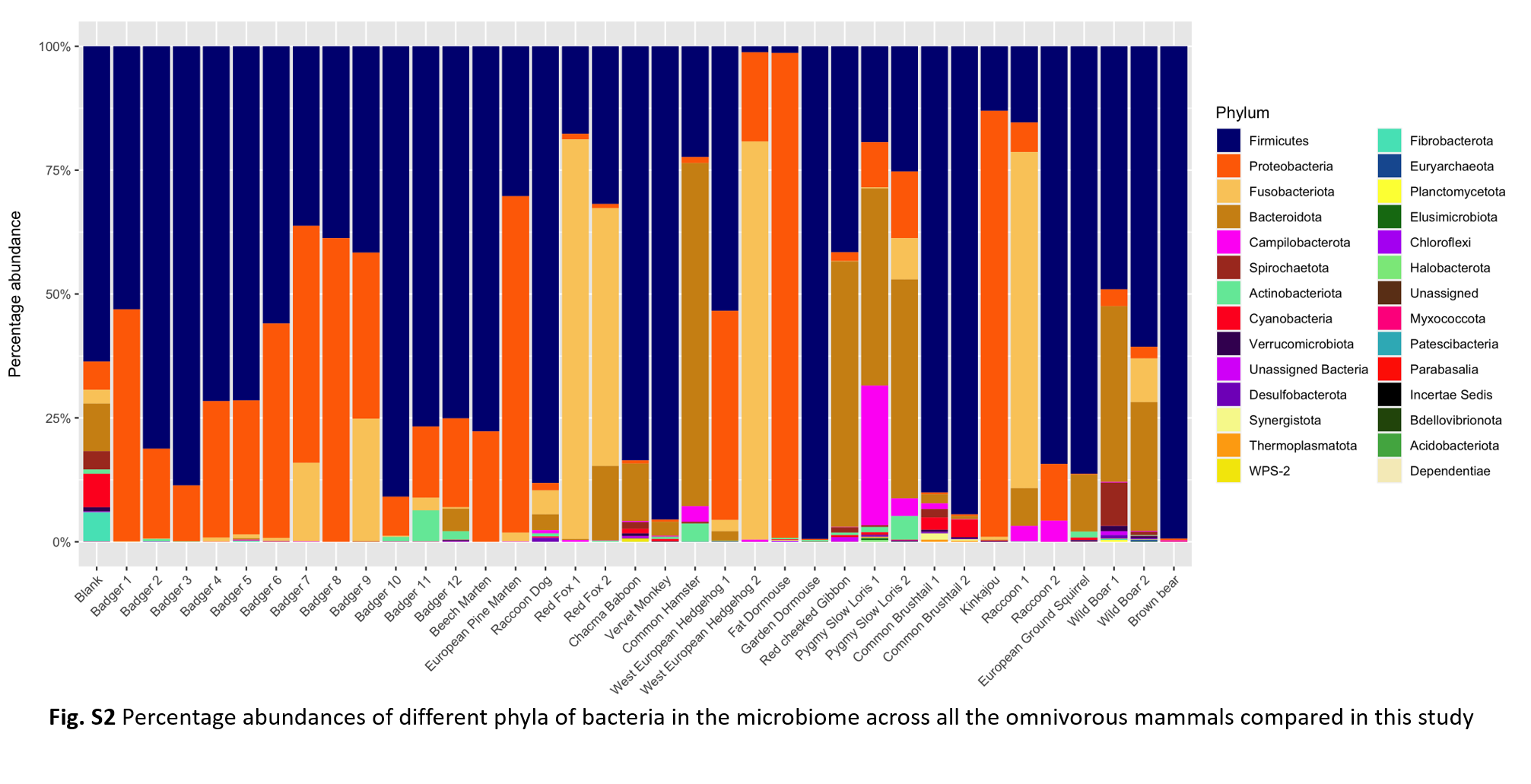

Supplement: Supplementary file 2 — Supplementary file2 (TIF 1137 kb) [file 284_2022_3064_MOESM2_ESM.tif]
